# Expression of the β3 subunit of Na^+^/K^+^-ATPase is increased in gastric cancer and regulates gastric cancer cell progression and prognosis via the PI3/AKT pathway

**DOI:** 10.18632/oncotarget.20894

**Published:** 2017-09-15

**Authors:** Li Li, Ru Feng, Qian Xu, Feiyue Zhang, Tong Liu, Jiang Cao, Sujuan Fei

**Affiliations:** ^1^ Department of Gastroenterology, The Affiliated Hospital of Xuzhou Medical University, Xuzhou, Jiangsu 221000, P.R. China; ^2^ Department of Gastroenterology, Xuzhou Medical University, Xuzhou, Jiangsu 221000, P.R. China; ^3^ Department of Hematology, The Affiliated Hospital of Xuzhou Medical University, Xuzhou, Jiangsu 221000, P.R. China

**Keywords:** ATP1B3, proliferation, apoptosis, migration, gastric cancer

## Abstract

ATP1B3 encodes the β3 subunit of Na^+^/K^+^-ATPase and is located in the q22-23 region of chromosome 3. Na^+^/K^+^-ATPase participates in normal cellular activities but also plays a crucial role in carcinogenesis. In the present study, we found that expression of the β3 subunit of Na^+^/K^+^-ATPase was increased in human gastric cancer tissues compared with that in normal matched tissues and that this increased expression predicted a poor outcome. ATP1B3 expression was elevated at both the mRNA and protein levels in gastric cancer cell lines relative to those in a normal gastric epithelial cell line. Interestingly, ATP1B3 knockdown significantly inhibited cell proliferation, colony-formation ability, migration, and invasion and increased apoptosis in human gastric carcinoma cell lines. Additionally, knockdown induced cell cycle arrest at the G2/M phase. Furthermore, we demonstrated that ATP1B3 silencing decreased the expression of phosphatidylinositol 3-kinase (PI3K), protein kinase B (AKT) and phosphorylated AKT (p-AKT), indicating that ATP1B3 regulates gastric cancer cell progression via the PI3K/AKT signalling pathway. Hence, the β3 subunit of Na^+^/K^+^-ATPase plays an essential role in the tumourigenesis of gastric cancer and may be a potential prognostic and therapeutic target for the treatment of gastric cancer.

## INTRODUCTION

Gastric carcinoma is one of the most common malignancies and the third leading cause of cancer-related deaths worldwide [1]. According to the cancer control programme of the World Health Organization, 7 million patients die of cancer worldwide each year, and 700,000 of these deaths are due to gastric cancer [2]. Despite improvements in detection and treatment, gastric carcinoma remains one of the most aggressive malignancies, with an extremely poor prognosis, and is a major cause of cancer death worldwide [3]. Most newly diagnosed patients present with incurable disease and have a median survival of less than 1 year [4]. Therefore, elucidation of the molecular mechanisms underlying gastric carcinoma is urgently needed to enhance early diagnosis and cure rates. New treatment approaches, such as targeted knockdown of oncogene expression have been assessed. Identification of critical targets in advanced stomach cancer is needed to develop effective treatments.

The Na^+^/K^+^-ATPase is a complex membrane protein that utilizes ATP to transport Na^+^ and K^+^ ions across plasma membranes; it is widely distributed in prokaryotic and eukaryotic cells. This enzyme is composed of three subunits, α, β, and a member of the FXYD family [5]. The classical functions of the Na^+^/K^+^-ATPase, such as the generation of a general membrane potential or its role in nutrient uptake, depend on its fully assembled form [6]. The association of different α and β subunit combinations creates diverse isoforms, leading to substantial physiological and pharmacological differences in ATP, Na^+^, and K^+^ affinities [7, 8]. Different subunits play various roles in human cell growth and development. The α subunit bears the Mg^2+^, Na^+^, K^+^ and ouabain-binding sites and is therefore considered the catalytic subunitt of the enzyme. The β subunit contains three isoforms β1, β2 and β3, and is involved in the structural and functional maturation of the holoenzyme [9, 10, 11]. The Na^+^/K^+^-ATPase is involved in various cellular activities; it maintains the ionic balance of the cell across the membrane, establishes high K^+^ and low Na^+^ in the cytoplasm, regulates the trans membrane potential and maintains the stability of the cellular environment. The ionic homeostasis maintained by this pump is vital for cell growth, differentiation, and survival [12]. This ion pump also modulates cell migration and cell-cell interactions. Na^+^/K^+^-ATPase not only participates in normal cellular activities but may also play a crucial role in tumourigenesis. Because Na^+^/K^+^-ATPase has multiple important roles, changes in Na^+^/K^+^-ATPase pump activity are associated with many diseases. Increased expression of Na^+^/K^+^-ATPase subunits was observed in gastric cancer [13], bladder cancer [14] and breast cancer [15]. Altering the expression and activity of Na^+^/K^+^-ATPase in cancer cells may affect cancer progression. Thus, Na^+^/K^+^-ATPase is a potential target for the development of anti-cancer drugs based on its role as a versatile signal transducer and its association with the development and progression of different cancers [16]. Knockdown of the Na^+^/K^+^-ATPase α1 subunit reduced non-small cell lung cancer (NSCLC) cell migration and proliferation [17]. Inhibition of the Na^+^/K^+^-ATPase α1 subunit inhibited HepG2 cell proliferation, induced apoptosis and led to S phase arrest [18]. The ATP1B3 gene, which encodes the β3 subunit of the Na^+^/K^+^-ATPase, is located in the q22-23 region of chromosome 3 [19–21]. Previous studies have demonstrated that ATP1B3 up-regulated lymphocyte activity and promoted the production of IFN-γ, IL-2, IL-4, and IL-10 [22, 23]. One study showed that ATP1B3 protein modulated HIV-1 restriction and NF-κB activation in a BST-2-dependent manner [24]. Although no research has demonstrated an association between ATP1B3 and gastric cancer, the well-known Na^+^/K^+^-ATPase inhibitors, including digoxin, ouabain, arenobufagin and bufalin, induced cell cycle arrest and apoptosis in many human cancer cells, including those derived from NSCLC [25], hepatoma [26] and pancreatic cancer [27]. A previous study demonstrated that the Na^+^/K^+^-ATPase inhibitor bufalin induced apoptosis of gastric cancer cells through the inhibition of the protein kinase B (AKT) signalling pathway via CBL-B and CBL-C [28]. Arenobufagin has been found to suppress adhesion, migration, and invasion and to induce apoptosis and autophagy via inhibition of the phosphatidylinositol 3-kinase (PI3K)/AKT/mammalian target of rapamycin (mTOR) pathway in human hepatoma cell lines [29]. Many studies have demonstrated that the PI3K/AKT signalling pathway regulates cancer cell proliferation, motility, survival and metabolism [30, 31]. Increasing evidence has shown that many cytotoxic drugs induce apoptosis in cancer cells by inhibiting the PI3K/AKT pathway [32, 33, 34]. However, whether ATP1B3 can mediate gastric cancer progression has not been determined, and the clinical relevance of ATP1B3 expression in gastric cancer is unclear. Moreover, the molecular mechanisms of ATP1B3 in gastric cancer cells have not yet been elucidated.

Therefore, we examined the biological effects of ATP1B3 knockdown in cancer cells for the first time. We verified the oncogenic function of ATP1B3 in gastric cancer and the correlation between ATP1B3 expression and clinicopathological features using immunohistochemical (IHC) staining. Furthermore, we examined the effect of ATP1B3 gene expression knockdown on the biological behaviour of gastric cancer cells using various assays. ATP1B3 expression was increased in gastric cancer samples, and knockdown of ATP1B3 expression inhibited gastric cancer cell proliferation, colony-formation ability, migration, and invasion and induced gastric cancer cell apoptosis via the PI3K/AKT pathway. Overall, our experiments demonstrated that ATP1B3 may be a potential therapeutic target in gastric cancer.

## RESULTS

### ATP1B3 expression in gastric cancer and normal tissues

**Figure 1:**
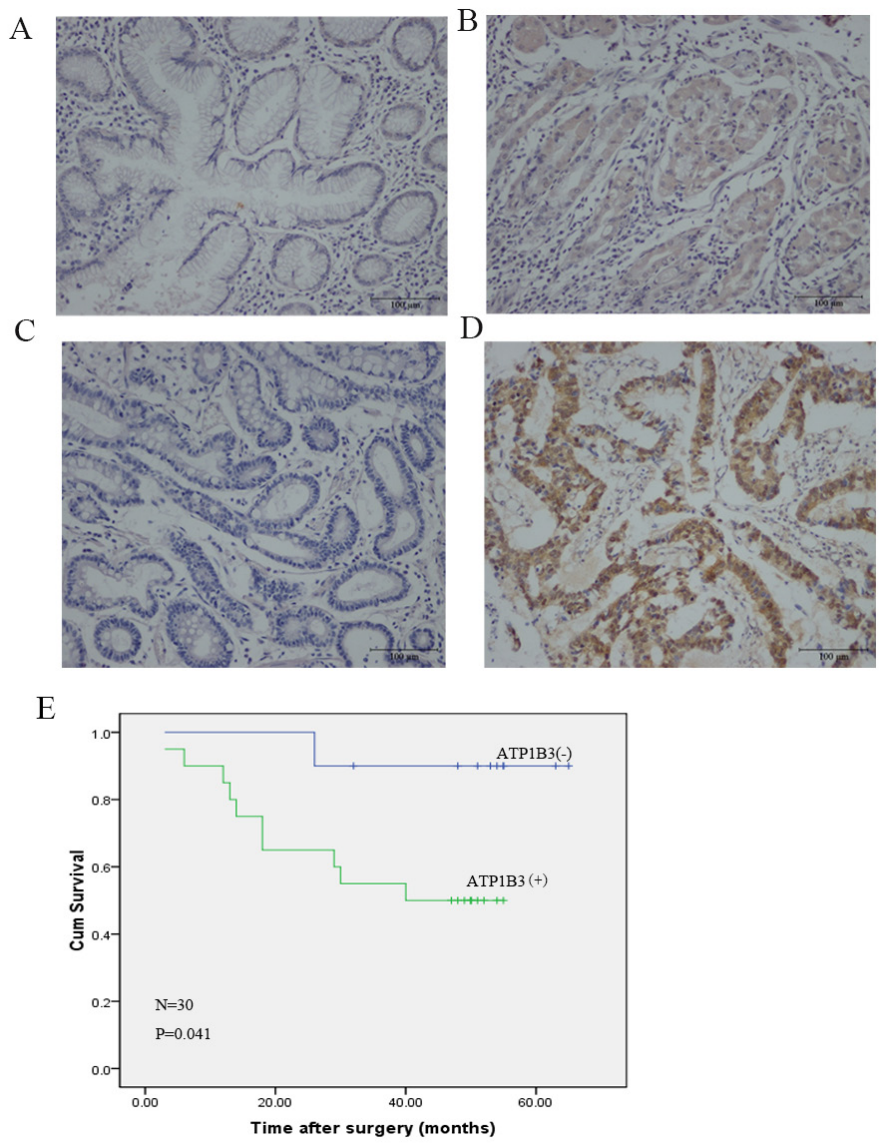
ATP1B3 expression is increased in human gastric cancer tissues Representative images of negative **(A)** and positive **(B)** ATP1B3 expression in adjacent normal tissues. Scale bar, 100 μm. Representative images of negative **(C)** and positive **(D)** ATP1B3 expression in gastric cancer tissues. Scale bar, 100 μm. **(E)** Relationship between ATP1B3 expression and gastric cancer prognosis. Kaplan-Meier curve of the overall survival of the patients with gastric cancer. The overall survival rate of gastric cancer patients with positive ATP1B3 expression was significantly lower than that of patients with negative expression (n=30, P=0.041). Therefore, these results indicated that ATP1B3 is increased inhuman gastric cancer and predicts a poor outcome.

To verify the oncogenic function of the ATP1B3 protein product in gastric cancer, we evaluated its expression in 30 primary gastric cancer tissue samples and matched normal tissue samples by IHC staining. The ATP1B3 protein was predominantly localized in cell membranes in both gastric cancer and normal tissues (Figure 1A-1D). ATP1B3 protein expression was higher in gastric cancer tissues than that in normal matched tissues. Positive ATP1B3 staining was detected in 66.7% (20/30) of the gastric cancer tissues and in only 36.7% (11/30) of the matched adjacent normal tissues (P<0.05) (Table 1). These data indicated that ATP1B3 protein expression is elevated in human gastric cancer tissues.

**Table 1:** ATP1B3 expression in gastric cancer and normal tissues

Tissue sample	n	Expression of ATP1B3		X^2^ value	P value
		Negative (n, %)	Positive (n, %)		
Gastric cancer	30	10(33.3)	20(66.7)	5.406	0.02
Normal tissue	30	19(63.3)	11(36.7)		

### The association between ATP1B3 expression and clinical characteristics

We investigated the correlation between ATP1B3 protein expression and clinicopathological features of 30 gastric cancer samples. As shown in Table 2, ATP1B3 expression was significantly associated with tumour invasion (T stage, P=0.015), lymph node metastasis (P=0.038) and cancer stage (AJCC, P=0.000). However, no significant correlation was found between ATP1B3 expression and sex, age, or degree of differentiation. ATP1B3 protein expression increased with further tumour development, suggesting that ATP1B3 protein up-regulation may be a relatively late event in gastric cancer. In a study of 30 patients, 20 patients demonstrated positive ATP1B3 protein expression, while the other patients demonstrated negative expression. With respect to the prognosis, these patients were followed-up until October 2016, and at that time, 19 patients were still alive and 11 had died; the minimum and maximum survival times were 3 months and 65 months after surgery, respectively. Based on the above findings, we used a univariate Kaplan-Meier analysis to assess the results of IHC staining and relevant clinical parameters in these previously studied thirty patients to clarify the prognostic value of increased ATP1B3 in gastric cancer. The overall survival rate of gastric cancer patients with positive ATP1B3 expression was significantly lower than that of patients with negative expression (n=30, X^2^=4.173, P<0.05) (Figure 1E). Together, these results indicated that ATP1B3 is increased inhuman gastric cancer and predicts a poor outcome.

**Table 2:** The association between ATP1B3 expression and clinical characteristics

Clinical characteristics	n	Expression of ATP1B3		X^2^ value	P value
		Negative	Positive		
Gender					
Male	16	7	9	3.662	0.056
Female	14	3	11		
Age					
≥60	14	6	8	0.840	0.359
<60	16	4	12		
Differentiation					
Well and moderate	13	5	8	0.271	0.602
Poor	17	5	12		
T stage					
T1+T2	4	4	0	5.867	0.015^*^
T3+T4	26	6	20		
N stage					
N0	6	4	2	4.311	0.038^*^
N1+N2	24	6	18		
Cancer stage					
I-II	11	8	3	12.129	0.000^*^
III-IV	19	2	17		

### ATP1B3 levels in gastric cancer cells

**Figure 2:**
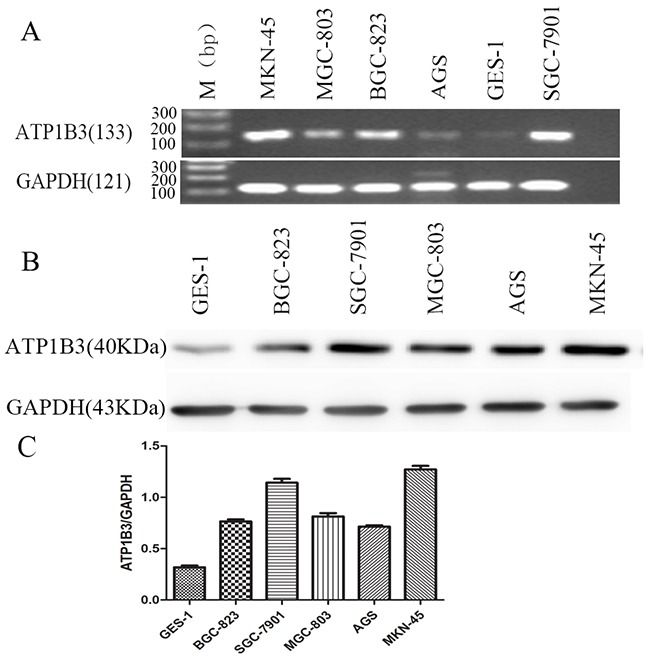
ATP1B3 mRNA and protein levels in gastric cancer cell lines **(A)** ATP1B3 mRNA expression levels in gastric cancer cell lines by RT-PCR. RT-PCR results showed that ATP1B3 was expressed in gastric cancer cells and GES-1 cells with different degrees of differentiation. **(B)** ATP1B3 protein expression levels in gastric cancer cell lines by Western blot. GAPDH was used as the endogenous control. M: Marker. ATP1B3 protein expression was increased in gastric cancer cells compared with normal gastric epithelial cells. **(C)** The relative integrated density values are expressed as the mean±SD. The results are representative of three independent experiments. Based on the average value, the expression level of ATP1B3 in SGC-7901 and MMKN-45 cells was higher.

We performed RT-PCR and Western blotting to measure ATP1B3 mRNA and protein levels, respectively, in the gastric cancer cell lines SGC-7901, MGC803, BGC-823, MKN-45, AGS and in the normal human gastric epithelial cell line GES-1. ATP1B3 mRNA was expressed in different gastric cancer cells and in GES-1 cells. ATP1B3 protein expression was increased in gastric cancer cells compared with that in normal gastric epithelial cells (Figure 2). Based on the average value, the expression level of ATP1B3 in SGC-7901 and MKN-45 cells was higher. Statistical analysis showed that the expression level of ATP1B3 in SGC-7901 and MKN-45 cells was significantly different from that of the other groups (P<0.05). SGC-7901 and MKN-45 cells displayed the highest endogenous expression of ATP1B3 among the gastric cancer cell lines examined. Therefore, we chose the moderately differentiated cell line SGC-7901 and the poorly differentiated cell line MKN-45 for further functional characterization.

### ATP1B3 expression was efficiently inhibited by siRNA in human gastric cancer cell lines

**Figure 3:**
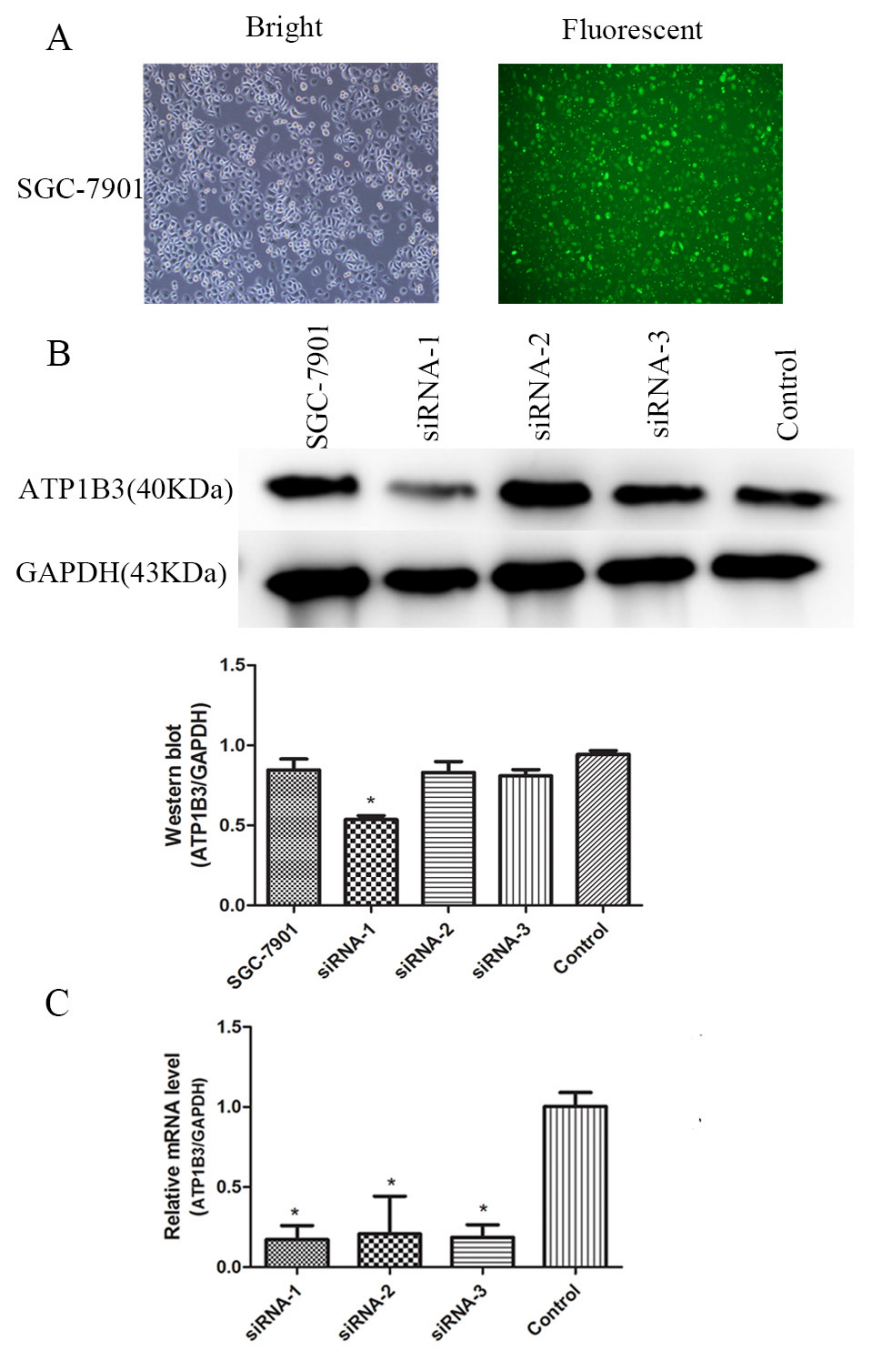
ATP1B3 expression was knocked down by transfection with ATP1B3-siRNA in SGC-7901 cells **(A)** Representative photomicrographs of bright fluorescence in SGC-7901 cells were obtained at a magnification of 100x. At 48 h after infection, approximately 50% of the cells expressed FAM at the time points as determined by fluorescence microscopy. **(B)** Western Blot measured ATP1B3 protein expression in SGC-7901 cells and in cells transfected with siRNA1, siRNA2, siRNA3, or si-NC. It showed that ATP1B3 siRNA1 was the most efficient knockdown sequence compared with the other siRNAs. ^*^P<0.05 compared with SGC-7901 and Control cells. **(C)** qRT-PCR assay analysis of ATP1B3 mRNA knockdown efficiency using siRNA1, siRNA2, siRNA3 and si-NC in SGC-7901 cells. ^*^P<0.0.1 vs. Control. All of three siRNA fragments demonstrated good knockdown efficiency with respect to ATP1B3 mRNA, and siRNA1 had the highest interference efficiency from the average value.

Next, we adopted siRNA to knockdown ATP1B3 expression in gastric cancer cells. To determine the transfection efficiency of siRNA, negative control-siRNA expressing FAM was transfected into SGC-7901 cells using Lipofectamine 2000. At 48 h after infection, approximately 50% of the cells expressed FAM as determined by fluorescence microscopy. We then examined the knockdown efficiency (Figure 3A). Western blotting and qRT-PCR were performed to evaluate ATP1B3 expression at both the protein and mRNA levels, respectively. Western blot analysis showed that of all the siRNAs, ATP1B3-siRNA1 was the most efficient knockdown sequence (Figure 3B). The qRT-PCR results indicated that the average knockdown efficiency of siRNA1, siRNA2 and siRNA3 was 82.8%, 79.3% and 81.5%, respectively (Figure 3C). Therefore, we transfected gastric cancer cells with ATP1B3-siRNA1 and ATP1B3-siRNA3 for the next set of experiments.

### The Na^+^/K^+^-ATPase activity of gastric cancer cells was knocked down by ATP1B3-siRNA

We divided the experimental cells into four groups: the Mock group (untreated cells), Experimental groups (the cells transfected with ATP1B3-siRNA1 and ATP1B3-siRNA3), and Control group (the cells transfected with non-specific siRNA). After transfection with ATP1B3-siRNA for 48 h, the Na^+^/K^+^-ATPase activity in the four groups of cells was detected according to the kit protocol. Finally, Na^+^/K^+^-ATPase activity was calculated according to an established formula. The unit of Na^+^/K^+^-ATPase activity is μmol Pi/mg/h. The results showed that Na^+^/K^+^-ATPase activity on the cell membrane was significantly reduced after down-regulation of ATP1B3 expression in SGC-7901 and MKN-45 cells. The differences between the levels of activity in the ATP1B3-siRNA1 and ATP1B3-siRNA3 groups and those in the Control group were statistically significant (P<0.01) in SGC-7901 and MKN-45 cells (Table 3). However, no significant difference was observed in Na^+^/K^+^-ATPase activity between the Mock and Control groups.

**Table 3:** The Na^+^ /K^+^-ATPase activity in each group of cells

Group	Mock	Control	ATP1B3-siRNA1	ATP1B3-siRNA3	P1	P3
SGC-7901	16.813±0.162	16.163±0.425	8.443±0.291	9.034±0.301	0.000	0.000
MKN-45	19.167±0.340	18.440±0.447	9.120±0.631	10.141±0.481	0.000	0000

### ATP1B3 knockdown suppressed the proliferation of gastric cancer cells

**Figure 4:**
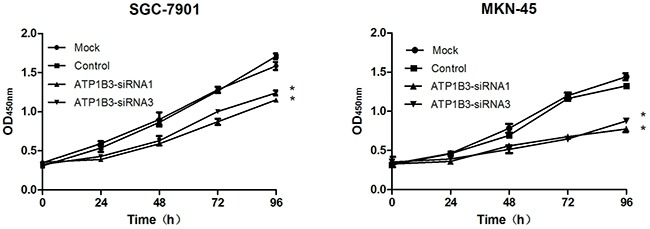
Knockdown of ATP1B3 led to the inhibition of SGC-7901 and MKN-45 cell proliferation Cells were cultured in 96-well plates and analysed by CCK-8 assay. Cell proliferation curves are shown after 96 hours. The absorbance value at a 450-nm wavelength in the ATP1B3-siRNA group was significantly lower than that in the Mock and Control groups. As time passed, the proliferation of ATP1B3-siRNA cells was more significantly inhibited. Mock, non-infected control cells; Control, cells infected with si-NC; ATP1B3-siRNA1, cells infected with ATP1B3-siRNA1; ATP1B3-siRNA3, cells infected with ATP1B3-siRNA3. ^*^P<0.05 vs. Control.

The effect of ATP1B3 knockdown on the proliferation of SGC-7901 and MKN-45 cells was determined by CCK-8 assays. During 96 h of observations, the CCK-8 assay results showed that the proliferation of SGC-7901 and MKN-45 cells was significantly reduced by ATP1B3 knockdown compared with that of the Mock and Control groups (P<0.05); however, no significant difference was observed between the Mock and Control groups. Thus, these data indicated that suppression of ATP1B3 in human gastric cancer cells inhibits cell proliferation *in vitro* (Figure 4).

### ATP1B3 knockdown inhibited gastric cancer cell colony-formation ability

**Figure 5:**
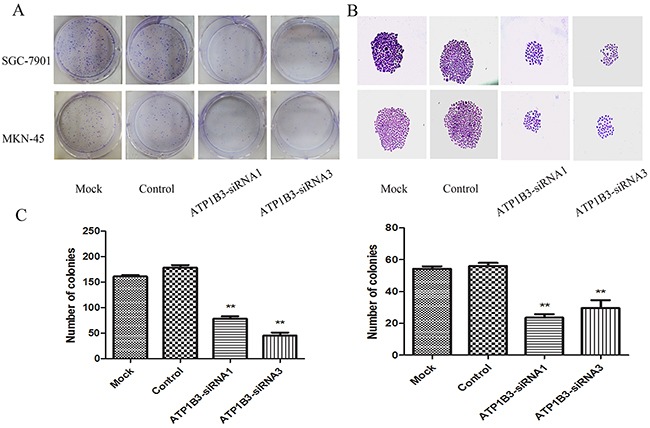
The effect of ATP1B3 silencing on the colony-formation ability of gastric cancer cells **(A)** Cells were cultured in 6-well plates and analysed by colony formation assay. After 14 days, the cells were stained, photographed and counted. Representative images of colonies formed by SGC-7901 and MKN-45 cells. **(B)** The size of the colonies in the ATP1B3-siRNA group was significantly smaller and the colonies were more dispersed than in the Mock and Control cell clone groups. **(C)** Statistical analysis of the numbers of SGC-7901 and MKN-45 cell colonies. The data are expressed as the mean±SD. The data are representative of three independent experiments. ^*^P<0.05 vs. Control. These results revealed that ATP1B3 knockdown inhibited the colony-forming ability of human gastric cancer cells.

To further evaluate proliferation, we assessed the colony-formation capacity of SGC-7901 and MKN-45 cells after treatment with ATP1B3-siRNA. Cells transfected with ATP1B3-siRNA1 and ATP1B3-siRNA3 or si-NC were incubated for 14 days to allow the formation of colonies. ATP1B3 knockdown resulted in significant decreases in the number of colonies in both SGC-7901 and MKN-45 cells (P<0.05) compared with those of the parental or control groups (Figure 5). These results revealed that ATP1B3 knockdown inhibited the colony-forming ability of human gastric cancer cells.

### Knockdown of ATP1B3 arrested cell cycle progression of gastric cancer cells

**Figure 6:**
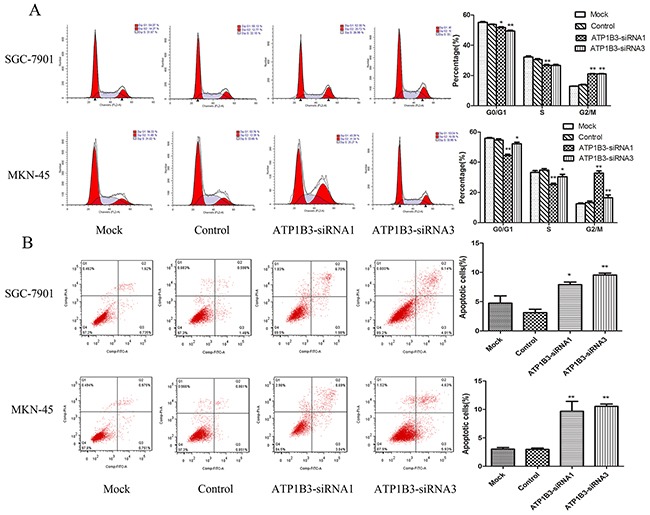
The effect of ATP1B3 knockdown on cell cycle and apoptosis detected by flow cytometry **(A)** After 48h of transfection with ATP1B3-siRNA, four groups of cells were collected to detect the cell cycle distribution. It was found that the percentage of ATP1B3-siRNA1 and ATP1B3-siRNA3 cells in G2/M phase was increased while the percentage of cells in G0/G1 and S phase was decreased compared with the Mock and Control groups of SGC-7901 and MKN-45 cells. Data represent the mean±SD of three independent experiments. ^*^P<0.05 vs. Control, ^**^P<0.01 vs. Control. Thus, the knockdown of ATP1B3 could arrest cell cycle progression of gastric cancer cells. **(B)** Down-regulation of ATP1B3 induced apoptosis of gastric cancer SGC-7901 and MKN-45 cells, as shown by flow cytometry. The number of apoptotic cells in the ATP1B3-siRNA1 and ATP1B3-siRNA3 group was significantly increased compared with that of the Mock and Control groups of SG -7901 and MKN-45 cells. ^*^P<0.05 vs. Control.

To investigate whether cell cycle arrest contributed to the cell proliferation and colony formation inhibition, we analysed the cell cycle of SGC-7901 and MKN-45 cells using flow cytometry after ATP1B3 knockdown. As shown in Figure 6A, knockdown of ATP1B3 arrested SGC-7901 and MKN-45 cells in G2/M phase and accordingly decreased the cell numbers in G0/G1 phase and S phase, suggesting that gastric cancer cells were arrested in G2/M phase after ATP1B3 knockdown.

### Down-regulation of ATP1B3 induced gastric cancer cell apoptosis

To further confirm the influence of ATP1B3 on gastric cancer cell apoptosis, we knocked down ATP1B3 in SGC-7901 and MKN-45 cells. Cell apoptosis was measured by AnnexinV-FITC/PI double-staining by FACS. The percentage of apoptotic SGC-7901 cells was significantly increased in the ATP1B3-siRNA group compared with that in the Control group (ATP1B3-siRNA1 7.56±1.30% vs. Control 3.12±1.01%, P=0.000; ATP1B3-siRNA3 9.54±0.562% vs. Control 3.12±1.01%, P=0.000). The percentage of apoptotic MKN-45 cells was also significantly increased in the ATP1B3-siRNA group compared with that in the control group (ATP1B3-siRNA1 9.67±3.00% vs. Control 3.10±2.44%, P=0.000; ATP1B3-siRNA3 10.54±0.685% vs. Control 3.10±2.44%, P=0.000), while the levels of apoptosis of the Mock group remained almost unchanged compared with the levels in the Control group (Figure 6B). These results confirmed that ATP1B3 affects apoptosis of gastric cancer cells.

### Influence of ATP1B3 silencing on the migration and invasiveness of gastric cancer cells

**Figure 7:**
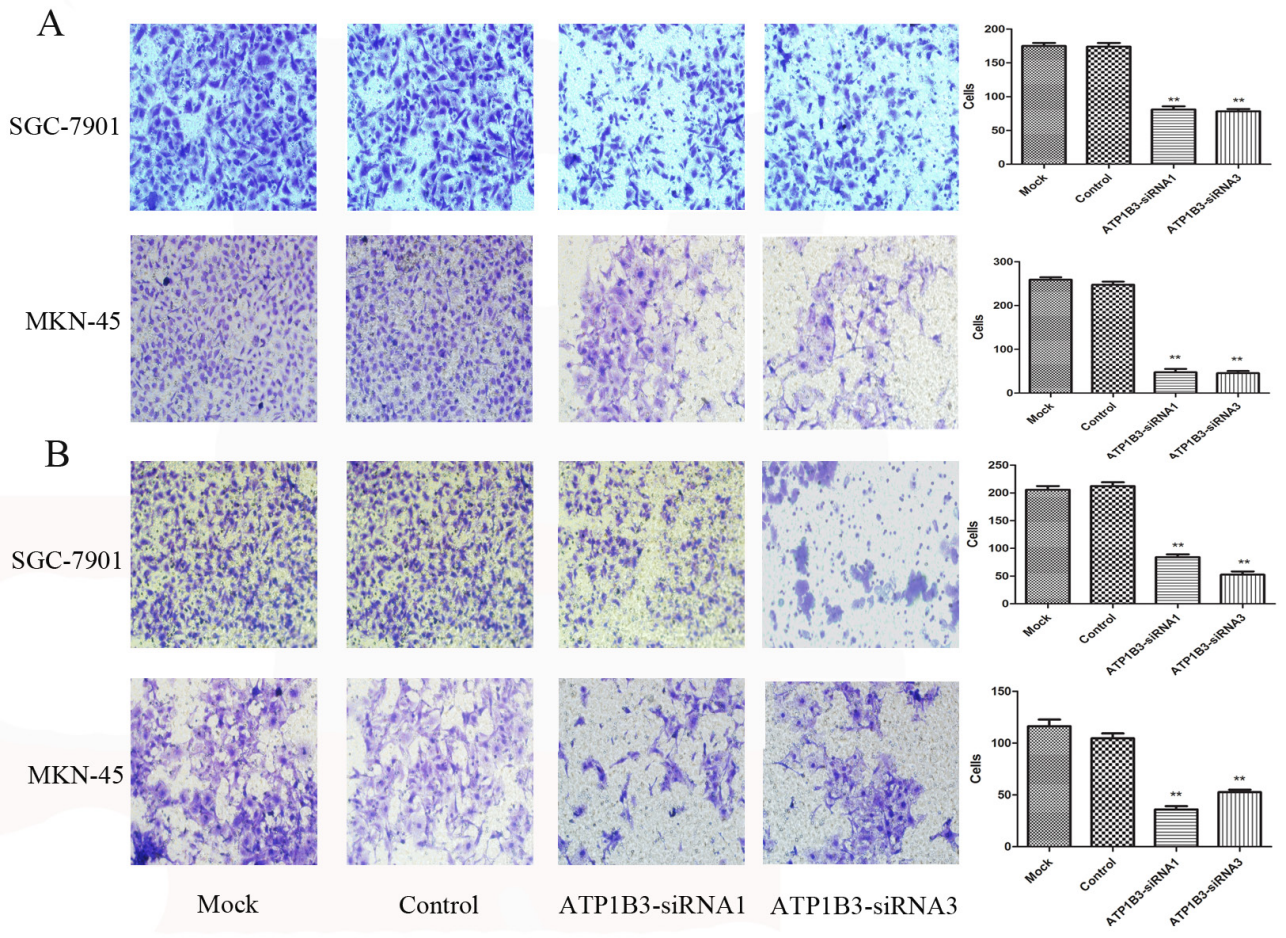
The effect of the knockdown of ATP1B3 expression on the migration and invasiveness of gastric cancer SGC-7901 and MKN-45 cells **(A)** Representative images and accompanying statistical plots are presented. The number of cells in the ATP1B3-siRNA1 and ATP1B3-siRNA3 group that migrated to the lower chamber was significantly less than that in other groups. Down-regulation of ATP1B3 in gastric cancer cells inhibited cell migration ability *in vitro*. ^**^P<0.01 vs. Control. The images were obtained at 200× magnification. **(B)** Invasion ability of the SGC-7901 and MKN-45 cells was suppressed in the ATP1B3-siRNA1 and ATP1B3-siRNA3 group compared with the Mock and Control groups according to a Transwell assay. Data are shown as the mean±SD. ^**^P<0.01 vs. Control. The images were obtained at 200× magnification.

Our cell proliferation and colony-formation assays demonstrated that suppression of ATP1B3 expression significantly reduced the proliferation of SGC-7901 and MKN-45 cells. Furthermore, to determine whether ATP1B3 knockdown inhibits cell migration, we employed Transwell chamber-based assays to elucidate the role of ATP1B3 in gastric cancer cell migration. As shown in Figure 7A, the numbers of ATP1B3-silenced cells on the surface of the Transwell membrane were lower than those of control and mock SGC-7901 and MKN-45 (P=0.000), while the differences in the number of SGC-7901 cells on the surface of the Transwell membrane compared with that in the Control group (P=0.882) and MKN-45 cells was not significant (P=0.245). Therefore, no significant difference was observed between the Mock and Control groups. These results indicated that ATP1B3 knockdown in SGC-7901 and MKN-45 cells led to a significant decrease in their migratory ability. To assess the effect of ATP1B3 silencing on the invasive ability of SGC-7901 and MKN-45 cells, we used *in vitro* Matrigel invasion assays. Cells transfected with ATP1B3-siRNA showed a more than 50% reduction in invasiveness of SGC-7901and MKN-45 cells compared with the Control and Mock groups (P=0.000) (Figure 7B). Together, these results indicated that ATP1B3 down-regulation in gastric cancer cells inhibits cell migration and invasion *in vitro*.

### ATP1B3 knockdown suppressed activation of the PI3K/AKT signalling pathway

**Figure 8:**
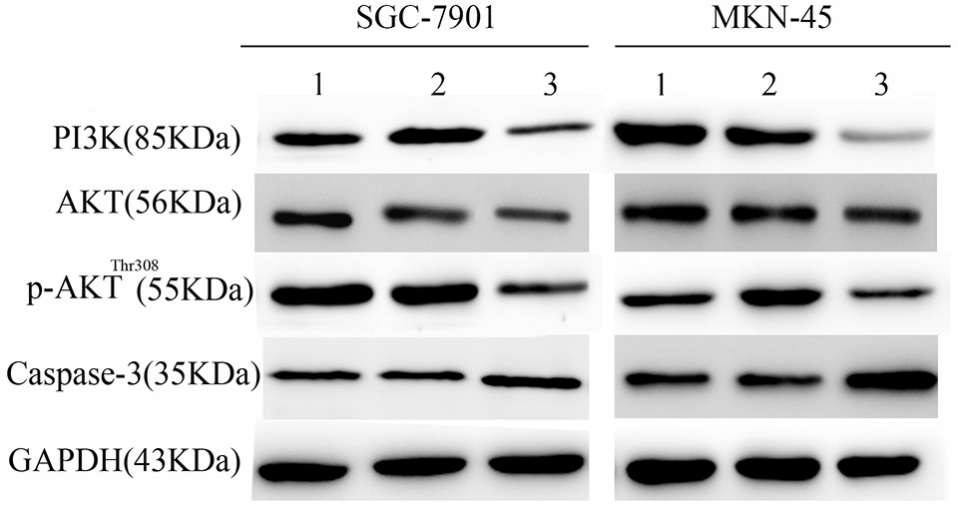
ATP1B3 knockdown suppressed activation of the PI3K/AKT signalling pathway The levels of PI3K, AKT, p-AKT and Caspase-3 protein in SGC-7901 and MKN-45 cells with altered expression of ATP1B3 were determined by Western blot.1 is the Mock group, 2 is the Control group, and 3 is the ATP1B3-siRNA group. Western blotting showed that knockdown of ATP1B3 resulted in the down-regulation of PI3K and AKT, attenuation of AKT phosphorylation, and activation of the apoptosis-related protein Caspase-3. GAPDH was used as an internal loading control. Each experiment was repeated three times, and similar results were obtained.

To explore the molecular mechanisms underlying ATP1B3-induced cell proliferation, migration and apoptosis, we investigated the effect of ATP1B3 on the PI3K/AKT signalling pathway in both SGC-7901 and MKN-45 cell lines by Western blot analysis. As shown in Figure 8, PI3K, AKT and phosphorylation of AKT were reduced in both gastric cancer cell lines after ATP1B3 knockdown. Altered expression of apoptosis markers, including Caspase-3, was detected in ATP1B3-silenced gastric cancer cells. Western blotting showed that knockdown of ATP1B3 promoted Caspase-3 activation. These results indicated that ATP1B3 knockdown inhibits gastric cancer cell growth and induced apoptosis via blockade of PI3K/AKT pathway activation.

## DISCUSSION

Recent studies have shown that changes in ATPase activity may be an underlying factor for tissue dysplasia, and Na^+^/K^+^-ATPase may be related to cancer cell metastasis. Na^+^/K^+^-ATPase has therefore been considered a potential target for oncotherapy. Current studies have predominantly focused on the function of the α subunit, but few have studied the β subunit. ATP1B3 encodes the Na^+^/K^+^-ATPase β subunit, an integral membrane protein essential for establishing and maintaining the Na^+^ and K^+^ electrochemical gradients across the plasma membrane. In addition, ATP1B3 was recently reported to be a novel determinant of pain variability [35] and impairment of the Na^+^/K^+^-ATPase β subunit increased the incidence of apoptosis among leukaemia cells [36, 37]; however, the complete oncogenic role of ATP1B3 in gastric cancer is still unclear.

In the present study, we investigated the association between ATP1B3 protein expression and gastric carcinogenesis and progression. Our clinical findings suggested that ATP1B3 protein is increased in gastric cancer compared with paired adjacent non-malignant tissues, which indicates that the β3 subunit of Na^+^/K^+^-ATPase may play an active role in the development of gastric cancer. Next, we investigated the clinicopathological relevance of ATP1B3 expression for gastric cancer patients. We observed that aberrant expression of ATP1B3 was associated with clinical stage. In addition, we also found a significant association between high ATP1B3 expression and worse clinical outcome, where patients with high ATP1B3 expression had shorter survival time than did patients with low ATP1B3 expression. These results suggest that ATP1B3 is a potential molecular target for gastric cancer therapy.

Consistent with previous studies, our results showed that ATP1B3 protein was increased in multiple gastric cancer cell lines compared with the normal GES-1 cell line. These findings may be related to tumour cell biological characteristics, namely, that the metabolism of tumour cells is more vigorous than that of normal cells; therefore, with a Na^+^/K^+^-ATPase level increase or enhancement, ATP1B3 expression increased accordingly. In our study, ablation of ATP1B3 inhibited cell proliferation, colony formation, migration and invasion and induced apoptosis in human gastric carcinoma cell lines, specifically in MKN-45 cells. Notably, ATP1B3 knockdown in gastric cancer cells resulted in G2/M phase arrest, which is consistent with the role of ATP1B3 in cell cycle progression. ATP1B3 silencing suppressed proliferation and progression of gastric cancer cells. These results indicated that ATP1B3 plays an important role in gastric cancer initiation, progression and invasive metastasis and may be a target for gastric cancer treatment. Hence, ATP1B3 may function as a pleiotropic modulator of gastric cancer progression, although the underlying mechanisms of ATP1B3 in gastric tumourigenesis need to be further elucidated. These results demonstrated that ATP1B3 plays an important role in gastric cancer cell proliferation, migration and invasion, suggesting an oncogenic role of ATP1B3 in gastric cancer.

The PI3K/AKT pathway is a key signalling pathway involved in multiple biological processes, such as cell proliferation, differentiation, death, migration, invasion and inflammation. The AKT signalling pathway plays an important regulatory role in many cellular survival pathways, primarily as an inhibitor of apoptosis; moreover, it is critical for angiogenesis and tumourigenesis [38]. Mechanistically, the PI3K/AKT signalling pathway is regarded as a key driver in carcinogenesis. PI3K activation triggers the recruitment of AKT and the phosphorylation of AKT to phospho-AKT [39]. Therefore, AKT is a key factor in the regulation of cell growth. Activated AKT may be present in the cytoplasm or may enter the nucleus to regulate anti-apoptotic proteins and cell proliferation [40]. In tumourigenic cells, PI3K can be activated by hormones, growth factors, and other stimuli. The activated PI3K produces the messenger PIP3, which promotes a conformational shift in inactive AKT [41]. We hypothesized that the PI3K/AKT signalling pathway may be involved in the regulation of ATP1B3 in gastric cancer cell proliferation and apoptosis. Western blot results suggested the PI3K/AKT signalling is significantly inactivated following ATP1B3 knockdown in gastric cancer cells. Meanwhile, in our study, down-regulation of ATP1B3 led to the activation of Caspase-3, which resulted in the induction of apoptosis.

In summary, the present study demonstrated that ATP1B3 is highly expressed in human gastric cancer tissues and cell lines and is closely correlated with the clinical features of patients with gastric cancer. ATP1B3 protein may be related to gastric tumourigenesis and tumour progression by affecting the PI3K/AKT signalling pathway. Therefore, increased ATP1B3 expression may be a useful marker for gastric cancer diagnosis and prognosis. In addition, ATP1B3 could be an effective potential therapeutic target for treatment of gastric cancer.

## MATERIALS AND METHODS

### Cell lines

The human gastric carcinoma cell lines MKN-45, SGC-7901, AGS, MGC-803 and BGC-823, as well as the normal human gastric epithelial cell line GES-1, were obtained from the Type Culture Collection of the Chinese Academy of Science (Shanghai, China). Cells were maintained in RPMI 1640 medium supplemented with 1% penicillin-streptomycin and 10% FBS (HyClone, Logan, UT, USA) at 37°C in 5% CO_2_.

### Human tissue samples

Tissue samples were obtained from 30 patients with gastric cancer who underwent tumour resection from May 2011 to December 2012 at the Affiliated Hospital of Xuzhou Medical University (Xuzhou, China).

The samples comprised paraffin-embedded primary neoplasms and matched non-tumour tissues (at least a 5 cm distance from primary neoplasms). None of the patients had been treated with chemotherapy or radiotherapy prior to surgery. This study was approved by the Ethics Committee of the Affiliated Hospital of Xuzhou Medical University. Informed consent according to the Declaration of Helsinkiwas obtained from each patient.

### IHC analysis

The slides containing the tissue samples were deparaffinized and rehydrated and then subjected to epitope retrieval according to routine methods. After endogenous peroxidase activity was blocked, the slides were incubated with 10% goat serum in PBS. The slides were then incubated with a primary rabbit monoclonal antibody against ATP1B3 (1:50; Abcam, ab137055, Shanghai, China) at 4°C overnight. The slides were washed three times with PBS and then incubated with an anti-rabbit secondary antibody for 2 h at 37°C. The tissue sections were stained with 3,3’-diaminobenzidine (ZSGB, Beijing, China) and haematoxylin. Finally, the slides underwent dehydration, clearing and mounting with neutral gums. The negative control samples were treated identically, but the rabbit antibody against ATP1B3 was replaced by PBS.

A combined scoring system, in which the staining intensity (SI) was multiplied by the percentage of positive cells (PP), was used to evaluate immunoreactivity for ATP1B3 protein expression. Scores from 0-3 were given for the SI or the PP, as follows: score of 0 (negative or < 5%); score of 1 (weak or 6-25%); score of 2 (moderate or 26-50%); score of 3 (strong or 51-70%); score of 4 (only 71-100%). Then, a final decision for ATP1B3 expression was made according to a standard scoring system (negative expression: score<4; expression: score≥4).

### ATP1B3-siRNA vector construction and cell transfection

Small interfering RNA (siRNA) targeting the humanATP1B3 gene (NM_001679.3), non-specific siRNA (si-NC) and negative control siRNA (FAM fluorescent tags) were designed as shown in Table 4. The siRNAs were synthesized by Gene Pharma (Shanghai, China).

**Table 4:** The siRNA sequences targeting the human ATP1B3 gene

Gene	Sequence (5′- 3′)
ATP1B3-siRNA1	5′-CAUUCACGAUGUGGGUUAUTT-3′
	5′-AUAACCCACAUCGUGAAUGTT-3′
ATP1B3-siRNA2	5′-GAAGAACAGAAGAACCUCATT-3′
	5′-UGAGGUUCUUCUGUUCUUCTT-3′
ATP1B3-siRNA3	5′-GGGACGAGUUAUGUUCAAATT-3′
	5′-UUUGAACAUAACUCGUCCCTT-3′
Negative control (FAM)	5′-UUCUCCGAACGUGUCACGUTT-3′
	5′-ACGUGACACGUUCGGAGAATT-3′

Cells were incubated in six-well plates at a density of 2×10^5^ cells/well. When the cell density reached 70-80%, the siRNAs were then transfected into SGC-7901 and MKN-45 cells using Lipofectamine 2000 according to the manufacturer's instructions (Invitrogen, California, USA). Infection efficiency was determined by counting the numbers of FAM-positive cells under a fluorescence microscope 48 h after infection. The knockdown efficiency of ATP1B3 was evaluated by real-time quantitative polymerase chain reaction (PCR) and Western blot analysis.

### Reverse-transcription PCR and real-time quantitative PCR

Total RNA was extracted from cultured cells using TRIzol reagent (Invitrogen, California, USA) and was synthesized into cDNA using M-MLV reverse transcriptase (Promega, Wisconsin, USA) according to the manufacturer's instructions. ATP1B3 mRNA expression was measured by real-time quantitative PCR using SYBR QPCR mastermix (Vazyme, Nanjing, China) in a TP800 Real-Time PCR machine (TaKaRa). The primers used were as follows: GAPDH forward,5’-TGAC TTCAACAGCCACACCCA-3’, GAPDH reverse, 5’-CAC CCTGTTGCTGTAGCCAAA -3’; ATP1B3 forward, 5’-AA CCCGACCACCGGAGAAAT-3’, and ATP1B3 reverse, 5’-TGAGAGTCTGAAGCATAACCCA-3’. GAPDH was used as an internal control for both reverse-transcription PCR and real-time quantitative PCR. PCR was performed with the following cycle profile: 94°C for 5 min; 94°C for 30 s, 53°C for 30 s, and 72°Cfor 35 s for 35 cycles, with a final extension step at 72°C for 2 min. The reverse-transcription PCR products were analysed by electrophoresis on a 2.0% agarose gel to compare the ATP1B3 mRNA levels among different cell lines. The relative mRNA expression was determined using the 2^-ΔΔCt^ method to calculate the ATP1B3 gene silencing efficiency. All samples were assayed in triplicate.

### Western blotting

Total protein was isolated from tissue samples or cultured cells using RIPA lysis buffer (Beyotime, Shanghai, China) with a protease inhibitor cocktail and was then quantified by BCA assay (Beyotime, Shanghai, China). Equal amounts (20 μg) of protein samples were loaded onto a 12% SDS-PAGE gel and then transferred to PVDF membranes. The membrane was blocked by 5% skim milk in PBS at room temperature for 1 h and incubated with primary rabbit antibodies against human ATP1B3 protein (1:1000; Abcam, ab137055, Shanghai, China), PI3K (1:1000; Abcam, ab189403, Shanghai, China), AKT (1:500; Abcam, ab8805, Shanghai, China), p-AKT (1:1000; Abcam, ab38449, Shanghai, China), Caspase-3(1:5000; Abcam, ab32351, Shanghai, China) and GAPDH (1:1000; Bioworld Technology, Beijing, China) overnight at 4°C. After the membranes were washed with TBST, they were incubated with horseradish peroxidase-conjugated goat anti-rabbit secondary antibody (1:2000; Abcam, ab6721, Shanghai, China) for 2 h at room temperature. Signals were detected by enhanced chemiluminescence (ECL) reagent (Biogot, USA).

### Assay of Na^+^/K^+^-ATPase activity

When the gastric cancer cells reached a logarithmic phase, they were centrifuged at 4°C for 5 minutes and washed three times with PBS. The collected cells were processed on ice using a homogenizer. The homogenates were then used to assay Na^+^/K^+^-ATPase activity. The activity of Na^+^/K^+^-ATPase was detected by phosphorus fixation in each group and was determined according to the kit protocol (Nanjing Jian cheng Bioengineering Institute, Nanjing, Jiangsu, China). After completion of the reaction, the 96-well plate was tested at a wavelength of 636 nm to detect the absorbance value. One unit (U) of Na^+^/K^+^-ATPase activity was defined as the amount of enzyme required to liberate 1 micromole of inorganic phosphate from the appropriate substrate per milligram protein per hour under the assay conditions.

### Cell proliferation assay

Gastric cancer cells (SGC-7901 and MKN-45) in the logarithmic phase after transfection with ATP1B3 siRNA were digested, resuspended, and inoculated in 96-well plates at a density of 5×10^3^ cells/well in a 5% CO_2_ incubator at 37°C. Then, 10 μl of CCK8 reagent (Keygen, Nanjing, China) was added to each well, and the cells were cultured for another 2 h at 37°C. The absorbance was measured at 450 nm using a micro-plate reader at 0 h, 24 h, 48 h, 72 h and 96 h. Cell growth curves were determined using the optical density value. Experiments were performed in triplicate.

### Colony-formation assay

After transfection, SGC-7901 and MKN-45 cells (500 cells/well) were seeded in six-well plates and incubated for 10 days to allow formation of normal colonies. The culture medium was replaced every 3 days. When the cell numbers in most single colonies were greater than 50, the cells were washed once with PBS and fixed in paraformaldehyde for 30 min at room temperature. The fixed cells were stained with freshly prepared Giemsa dye for 20 min. After the samples were washed several times with ddH_2_O, the number of colonies was counted under a light microscope. Each experiment was performed at least in triplicate.

### Cell cycle analysis

Cells were cultured in 6-well plates and were transfected with ATP1B3-siRNA or Control siRNA. When the cells reached 80% confluence, they were collected and fixed in 70% ice-cold ethanol for at least 1 h. Cells were then washed with PBS and resuspended in 500 μl PBS containing 50 μg/ml propidium iodide (PI) and 100 μg/ml protease-free RNase. The suspension was incubated in the dark at room temperature for 30 min and then run in a FACS Calibur Flow Cytometer (BD Biosciences, USA). Data were analysed with the ModFit DNA analysis program.

### Flow cytometry apoptosis assay

After 48 h of transfection with ATP1B3-siRNA or Control siRNA, the cells were harvested and washed with binding buffer, resuspended in staining buffer, and then incubated with Annexin V-PI (BD, USA) at room temperature in the dark for 15 min. After incubation, the cells were analysed using a FACS Calibur Flow Cytometer (BD Biosciences, USA). All experiments were performed in triplicate.

### Cell migration and invasion assays

Migration assays were performed using Transwell membranes (Corning, New York, USA). After 48 h of infection, the cells (1×10^5^) were plated in the upper chamber with serum-free medium, while the lower compartment was filled with 20% FBS as a chemo attractant. After incubation for 24h, the cells that remained in the upper chamber were carefully removed with cotton swabs, while the cells that had migrated were fixed in 3% paraformaldehyde and stained with crystal violet. Cells in 5 independent 20x fields per well were then counted and imaged under a microscope.

For the invasion assay, prior to the inoculation of cells in the Transwell upper chamber, Transwell membranes were coated with Matrigel (BD-Bio, USA). Otherwise, the experimental process was performed as described above.

### Statistical analyses

All statistical analyses were performed using SPSS 19.0 software (SPSS, Inc., Chicago, IL, USA). Student's *t* test was used to compare means between two groups. The statistical data for each group are presented as the mean±SD. A value of P<0.05 was defined as significant.
